# Endothelial colony forming cells and mesenchymal progenitor cells form blood vessels and increase blood flow in ischemic muscle

**DOI:** 10.1038/s41598-017-00809-1

**Published:** 2017-04-10

**Authors:** Kyu-Tae Kang, Ruei-Zeng Lin, David Kuppermann, Juan M. Melero-Martin, Joyce Bischoff

**Affiliations:** 1 Vascular Biology Program and Department of Surgery, Boston Children’s Hospital, Harvard Medical School, Boston, MA 02115 USA; 2 College of Pharmacy and Innovative Drug Center, Duksung Women’s University, Seoul, Republic of Korea; 3 Department of Cardiac Surgery, Boston Children’s Hospital, Harvard Medical School, Boston, MA 02115 USA; 4 Harvard Medical School, Boston, MA USA; 5 College of Pharmacy and Innovative Drug Center, Duksung Women’s University, Pharmacy building (Room 423), 33, Samyangro 144-gil, Dobong Gu, Seoul South Korea

## Abstract

Here we investigated whether endothelial colony forming cells (ECFC) and mesenchymal progenitor cells (MPC) form vascular networks and restore blood flow in ischemic skeletal muscle, and whether host myeloid cells play a role. ECFC + MPC, ECFC alone, MPC alone, or vehicle alone were injected into the hind limb ischemic muscle one day after ligation of femoral artery and vein. At day 5, hind limbs injected with ECFC + MPC showed greater blood flow recovery compared with ECFC, MPC, or vehicle. Tail vein injection of human endothelial specific *Ulex europaeus agglutinin*-*I* demonstrated an increased number of perfused human vessels in ECFC + MPC compared with ECFC. *In vivo* bioluminescence imaging showed ECFC persisted for 14 days in ECFC + MPC-injected hind limbs. Flow cytometric analysis of ischemic muscles at day 2 revealed increased myeloid lineage cells in ECFC + MPC-injected muscles compared to vehicle-injected muscles. Neutrophils declined by day 7, while the number of myeloid cells, macrophages, and monocytes did not. Systemic myeloid cell depletion with anti-Gr-1 antibody blocked the improved blood flow observed with ECFC + MPC and reduced ECFC and MPC retention. Our data suggest that ECFC + MPC delivery could be used to reestablish blood flow in ischemic tissues, and this may be enhanced by coordinated recruitment of host myeloid cells.

## Introduction

Peripheral arterial disease (PAD) is an indication of systemic atherosclerosis that is undertreated in the United States, and is present in 29% of people over the age of 70 and prevalent in those over the age of 50 with a history of smoking and/or diabetes. PAD is characterized by the occlusion of blood vessels, and its progression results in ischemic ulceration and gangrene, leading to amputation in more than a third of patients. Thus, building new vascular networks to reestablish blood perfusion is one of the therapeutic goals to treat ischemic vascular diseases such as critical limb ischemia, stroke, and myocardial infarction.

Many different approaches to generate vascular networks have been pursued to stimulate recovery of blood perfusion within ischemic tissues. Angiogenic factors have been delivered by gene therapy or protein delivery to promote angiogenesis, yet clinical trials to date have not been successful. Building vascular networks using stem and progenitor cells from different sources has emerged as a new approach. Autologous adult stem/progenitor cells rather than embryonic stem cells have been a preferred strategy to achieve *de novo* vascularization in order to avoid the risks of teratoma formation^1^ and host immune response to allogeneic embryonic stem cells^2^.

We demonstrated that a “two cell strategy” – consisting of human endothelial colony forming cells (ECFC) and human mesenchymal progenitor cells (MPC) - can be used to form perfused human blood vessels in immune-deficient mice^3^. ECFC, also called late endothelial progenitor cells (EPC), and MPC form vascular networks *in vivo* when implanted in a variety of extracellular matrices^4, 5^. Furthermore, the newly formed human vascular networks can be transplanted to other sites; this demonstrates the nascent human vessels have an ability to reconnect with neighboring vasculature^6^. This versatility led us to propose that ECFC and MPC would form neo-vessels that integrate with existing host vessels in ischemic sites and thereby reestablish and improve blood perfusion within ischemic tissues.

The pro-angiogenic features of subpopulations of peripheral blood mononuclear cells (MNCs) have been described^7, 8^. Clinical and experimental reports have shown that infiltrated accessory myeloid cells, including monocytes, macrophages, neutrophils, eosinophils, mast cells and dendritic cells actively contribute to pathological neovascularization^9–14^. Myeloid cells have been shown to contribute neo-vessel formation by paracrine mechanisms when recruited to perivascular sites of neovascularization^15^. Neutrophil-derived matrix metalloproteinases (MMP)-2 and -9^16^ and/or myeloid cell-derived VEGF-A^17–19^ have been shown to play critical roles in blood vessel formation and growth. In other studies, subpopulations of myeloid cells were observed at the tips of nascent capillaries in the neonatal murine retina^20^ and in growth factor-induced angiogenesis and tissue regenerating regions^21–24^, suggesting that myeloid cells provide physical support to the vascular sprouting process. However, few studies have been done to ascertain the role of myeloid cells when vasculogenic cells such as ECFC and MPC are injected for therapeutic blood vessel regeneration in ischemic tissues.

In the present study, we investigated whether ECFC and MPC form vascular networks and restore blood flow in ischemic skeletal muscle, compared to ECFC or MPC alone, and whether host myeloid cells play a role. Our results indicate that ECFC + MPC delivery provides rapid recovery of blood flow in ischemic tissues by stimulating formation of new vessels, and that host myeloid cells play a pivotal role.

## Results

### ECFC + MPC improve blood flow recovery in ischemic hind limb muscles

**Figure 1:**
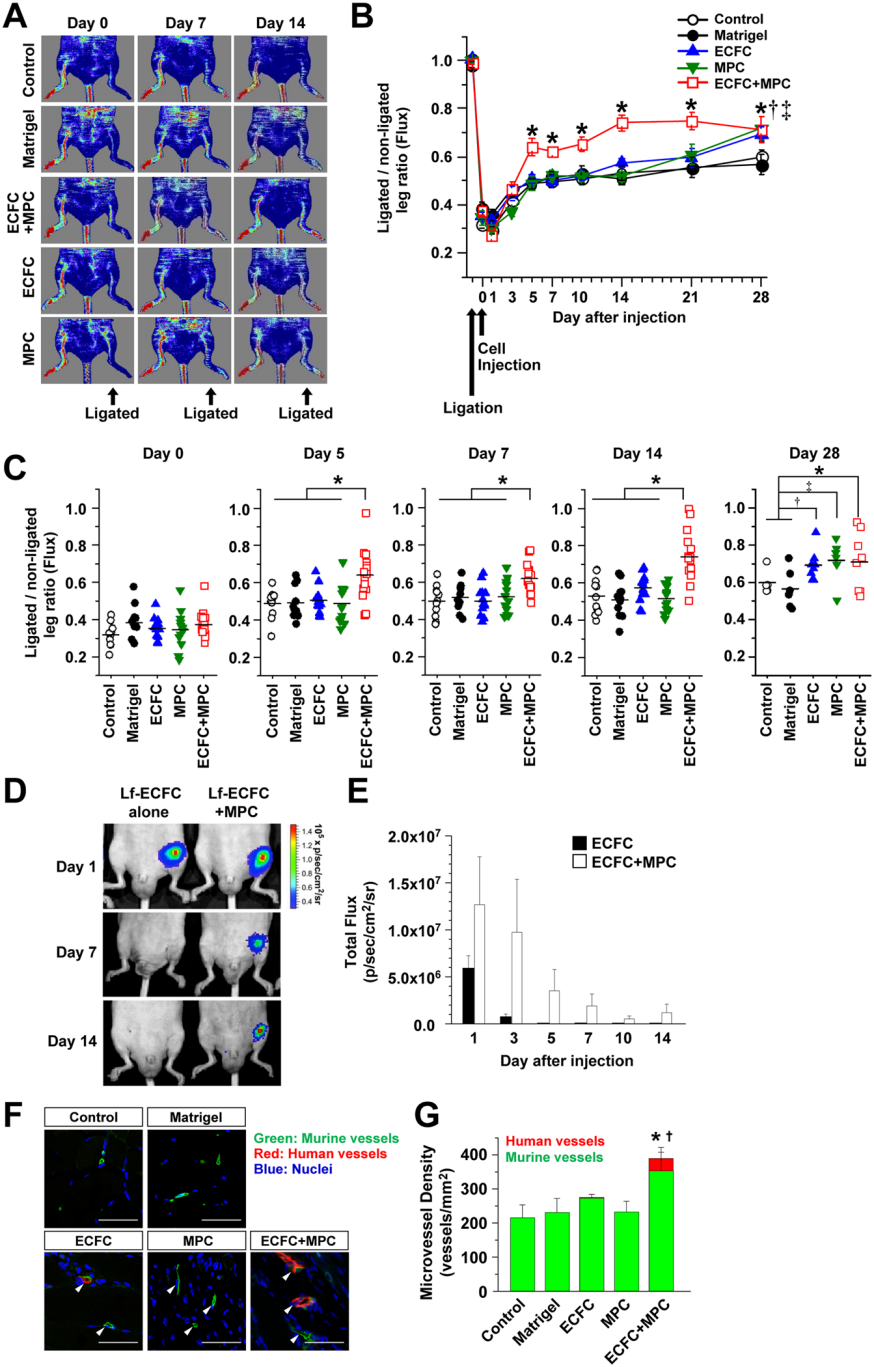
Beneficial effect of human ECFC + MPC on blood flow recovery in the murine ischemic hind limb muscles. Hind limb ischemia was induced by the ligation and cutting of femoral artery and vein at day -1. ECFC, MPC, or ECFC + MPC suspended in Matrigel were injected on day 0. Blood flow was measured by the Laser Doppler imager. Retention of luciferase-labeled ECFC was measured by bioluminescence using the Xenogen IVIS *in vivo* imaging system. At day 14, perfused human and rat vessels were identified by tail-vein injection of a mixture of rhodamine (red)-conjugated UEA I and FITC (green)-conjugated GS-IB_4_. (**A**) Representative laser Doppler images of ischemic hind limbs with/without cell injection over 14 days. (**B**) Graph of blood flow presented as the ligated/non-ligated leg ratio (n = 11–15; means ± SEM.). *Significant difference (P ≤ 0.05) between groups. (**C**) Dot graph expressed as single values for each mouse (n = 6–15; means shown by horizontal bars). *Significant difference (P ≤ 0.05) between groups. (**D**) Representative bioluminescence images of mice after injection of luciferase-labeled ECFC alone or luciferase-labeled ECFC + MPC. (**E**) Graph of bioluminescence signals detected in ischemic hind limbs (n = 3–4; means ± SEM.). (**F**) Representative confocal images of lectin-labeled vessels in the ischemic hind limb muscle at day 14 (Scale bars represent 50 μm). White arrowheads point to lectin-labeled (i.e. perfused) vessels. (**G**) Graph of total, human plus murine microvessels, in the ischemic hind limb muscles with/without cell injections (n = 3; means ± SEM.). *Significant difference (P ≤ 0.05) between groups for total microvessel density. ^†^Significant difference (P ≤ 0.05) between groups for human microvessel density.

Hind limb ischemia was induced by ligation, followed by cutting of femoral artery and vein. Blood flow was reduced to 31.94 ± 1.82% compared to the contra-lateral non-ligated leg at day 1, and recovered spontaneously to 53.10 ± 2.94% by day 14 in the control group without any treatment. To represent a potential clinical application, we injected ECFC + MPC suspended in Matrigel into the ischemic hind limb muscle one day after the hind limb ischemia induction. ECFC + MPC injection improved blood flow significantly at day 5 (64.05 ± 3.53 vs. 48.70 ± 2.30%, p < 0.05) and improved blood flow was maintained through day 21 compared to control, Matrigel, ECFC, or MPC injections (Fig. 1A,B,C). Figure 1C provides a visual representation of blood flow recovery for each animal in each treatment group at each time point. At 28 days, injection of ECFC + MPC, ECFC alone or MPC alone all showed significant improvement in blood flow recovery compared to no treatment (control) or Matrigel injection (Fig. 1B and C). In another experiment, the injected cell number was doubled; this did not result in additional improvement of blood flow recovery (Supplementary Fig. 1). These results demonstrate that injection of ECFC + MPC achieves significantly increased blood flow as early as day 5.

We next used *in vivo* bioluminescence imaging to assess retention and localization of the injected ECFC. Luciferase-labeled ECFC were injected alone or in combination with MPC into ischemic hind limb muscle. The bioluminescence signal was generated by systemic administration of the luciferase substrate luciferin. A linear correlation between bioluminescence signal and ECFC number was confirmed using serially diluted luciferase-labeled ECFC lysates (Supplementary Fig. 2A,B). The bioluminescence signal persisted for 14 days in ischemic hind limbs injected with ECFC + MPC (Fig. 1D,E). In contrast, the signal from hind limbs injected with luciferase-labeled ECFC alone was lost after day 3 (Fig. 1D,E). The persistent bioluminescence signal in the ECFC + MPC-injected hind limbs suggests that MPC support cellular retention of co-injected ECFC in ischemic tissue and perhaps vascular incorporation as shown previously^25^. The transient signals at day 1 and 3 observed when ECFC alone were injected may be due leakage of luciferin into vessels damaged during the femoral artery and vein ligation and cell injection procedures.

### ECFC + MPC form vessels in ischemic hind limb muscles

We hypothesized that improved blood flow recovery with ECFC + MPC injection is due to formation of new vessels. To support this, human ECFC-lined perfused vessels in ischemic hind limb were directly visualized by *in vivo* labeling: tail vein injection of a mixture of rhodamine-conjugated *Ulex europaeus agglutinin*-*I* (UEA I), a lectin specific for human endothelium, and FITC-conjugated *Griffonia simplifolia* isolectin B4 (GS-IB4), a lectin specific for rodent endothelium^6^. The advantages of this *in vivo* labeling technique are to (i) identify functional perfused vessels in contrast to dead-end tubular structures, (ii) distinguish human from murine vessels, and (iii) visualize the site of anastomosis. Three distinct patterns were observed: UEAI-positive human vessels (red), GS-IB4-positive murine vessels (green), and UEA I/GS-IB4- positive chimeric vessels (Fig. 1F). The total number of perfused vessels (both red and green fluorescent vessels) was significantly higher at day 14 in ischemic hind limb muscles injected with ECFC + MPC compared to other treatments (Fig. 1G). Perfused human vessels (UEA I-labeled red fluorescent vessels) were detected in ECFC + MPC and ECFC alone, but not in MPC alone, Matrigel, or control groups. Importantly, the number of human vessels was significantly higher in ECFC + MPC than ECFC alone (36.66 ± 22.17/mm^2^ vs. 2.90 ± 2.90/mm^2^, p < 0.05). The number of murine vessels (GS-IB4-labeled green fluorescent vessels) was significantly higher in ECFC + MPC group compared to control group (352.90 ± 54.88/mm^2^ vs. 215.20 ± 38.48/mm^2^, p < 0.05; Fig. 1G). ECFC alone, MPC alone, or Matrigel group did not show an increase in the number of perfused murine vessels compared to control group. This experiment shows that ECFC form the endothelial lining of some perfused blood vessels at the injection site when co-injected with MPC. Furthermore, ECFC + MPC injection increased the number of perfused murine vessels at the cell injection site. Images shown in Supplementary Fig. 2C show the new human vessels located in between muscle fibers.

### Host myeloid cells recruited to ECFC + MPC-injected ischemic hind limbs

We previously showed that injection of ECFC + MPC subcutaneously into mice leads to a transient influx of host CD11b^+^ myeloid cells into the Matrigel implant^26^. The influx starting at day 2 suggested myeloid cells play an active role in processes that precede the formation of functional vessels by ECFC + MPC. To determine whether host myeloid cells play a role when ECFC + MPC form vessels in a pathological ischemic tissue, we performed immunofluorescent and flow cytometric analyses to detect and quantify myeloid cells in the ischemic hind limb muscles in the absence and presence of ECFC + MPC injection.

**Figure 2:**
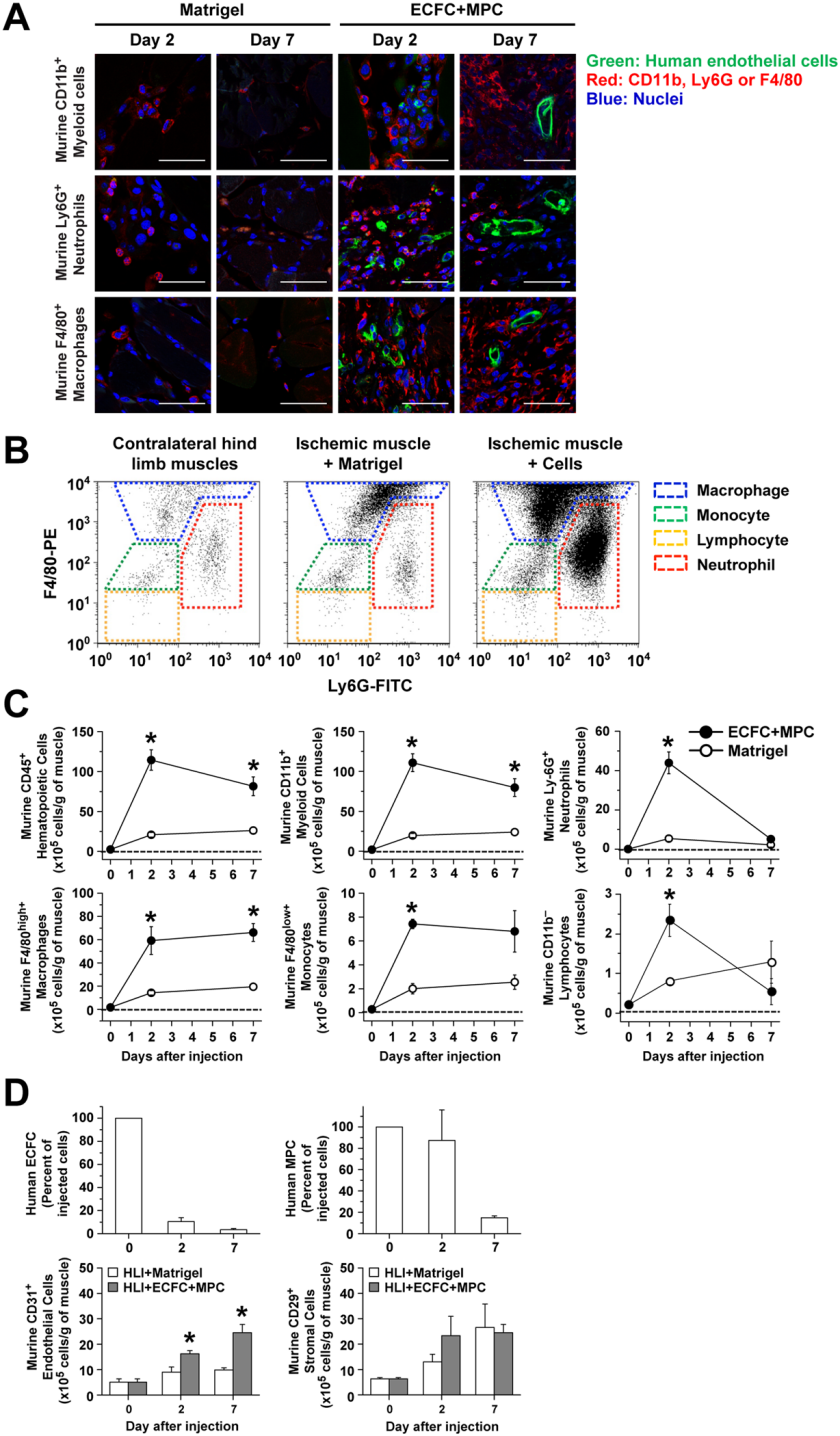
Myeloid cells recruited into murine ischemic hind limb muscle after human ECFC + MPC injection. Ischemic hind limb muscles with/without cell injection were harvested, fixed, and sectioned for confocal microscopy analysis. Sections were incubated with biotinylated UEA I followed by Fluorescein-Streptavidin to detect human ECFC (green). After that, sections were incubated with primary antibodies (rat anti-mouse CD11b, rat anti-mouse Ly-6G, or rat anti-mouse F4/80 antibodies) followed by goat anti-rat Alexa Fluor-568 (red). In parallel experimental sets, ischemic hind limb muscles were harvested, digested and analyzed by flow cytometry. (**A**) Representative confocal images of myeloid cells in the ischemic hind limb muscles injected with Matrigel or ECFC + MPC at day 2 and 7 (scale bars represent 50 μm). (**B**) Representative flow cytometry analyses of contralateral hind limb muscles and ischemic hind limb muscles injected with Matrigel or ECFC + MPC at day 2. (**C**) Quantitative cytometric analyses of myeloid lineage cells obtained from ischemic hind limb muscles (n = 3; means ± SEM). ^○^Shows cell number obtained from ischemic hind limb muscles injected with Matrigel alone. ^●^Shows cell number obtained from ischemic hind limb muscles injected with ECFC + MPC in Matrigel. *Significant difference (P ≤ 0.05) between groups. (**D**) Quantitative cytometric analyses of human and murine vascular cells and murine stromal cells obtained from ischemic hind limb muscles at day 0, 2, and 7 (n = 3; means ± SEM). *Significant difference (P ≤ 0.05) between groups.

The dynamic recruitment of CD11b^+^ myeloid cells, Ly-6G^+^ neutrophils, and F4/80 + macrophages into the ischemic hind limb muscles was observed by immunofluorescence (Fig. 2A). Cells expressing CD11b, Ly-6G, or F4/80 were abundant at day 2 in ECFC + MPC injected muscles compared to Matrigel injected. Flow cytometry of cells obtained from digested muscle was performed to quantify myeloid cells within the ECFC + MPC injection site. First, we analyzed levels of each myeloid lineage in hind limb muscle after ischemic induction. CD45^+^ hematopoietic cells, CD11b^+^ myeloid cells and myeloid lineage cells including Ly-6G^+^ neutrophils, F4/80^high+^ macrophages, and F4/80^l^°^w+^ monocytes as well as CD11b^−^ lymphocytes were increased at day 2 in the ischemic hind limb muscles without any treatment compared to contra-lateral hind limb muscles (Supplementary Fig. 3A); increased cell numbers were maintained until day 7 (data not shown). Interestingly, ECFC + MPC injection caused a significant increased in all myeloid lineage cell types at day 2 compared to Matrigel injection, suggesting that ECFC + MPC enhances myeloid cell recruitment (Fig. 2B,C). CD11b^+^ myeloid cells and F4/80^high+^ macrophages were increased significantly at day 2 and 7 in ischemic hind limb muscles with ECFC + MPC injection compared to Matrigel injection (Fig. 2C). Ly-6G^+^ neutrophils and F4/80^low+^ monocytes in hind limbs with ECFC + MPC injection were increased significantly at day 2 but were not different at day 7 compared to Matrigel injection. In peripheral blood, there were no differences in numbers of myeloid lineage cells between ECFC + MPC and Matrigel injection (Supplementary Fig. 3B), indicating that the increase in myeloid cells in ischemic hind limb muscles was due to the local ECFC + MPC injection.

Flow cytometric analysis was also performed to determine the retention rate of ECFC and MPC in the ischemic hind limb muscles. The number of ECFC and MPC decreased in a time dependent manner, but cells were detected on day 7 (Fig. 2D and Supplemental Table 1). At day 7, the number of ECFC was 0.50 × 10^5^ (6.25% of injected cells), and the number of MPC was 2.00 × 10^5^ (16.67% of injected cells). Murine CD31^+^ endothelial cells, but not CD29^+^ stromal cells, were higher at day 2 and 7 in the ECFC + MPC-injected hind limb muscles compared to Matrigel-injected hind limb muscle (Fig. 2D). These results suggest that, even though retention is low, grafted ECFC and MPC form functional blood vessels as well as stimulate host endothelial cells, which is well correlated with the increase in both human and murine vessels in ECFC + MPC-injected ischemic hind limb muscles (Fig. 1G).

### Host myeloid cells contribute to improved blood flow in ECFC + MPC-injected ischemic hind limbs

**Figure 3:**
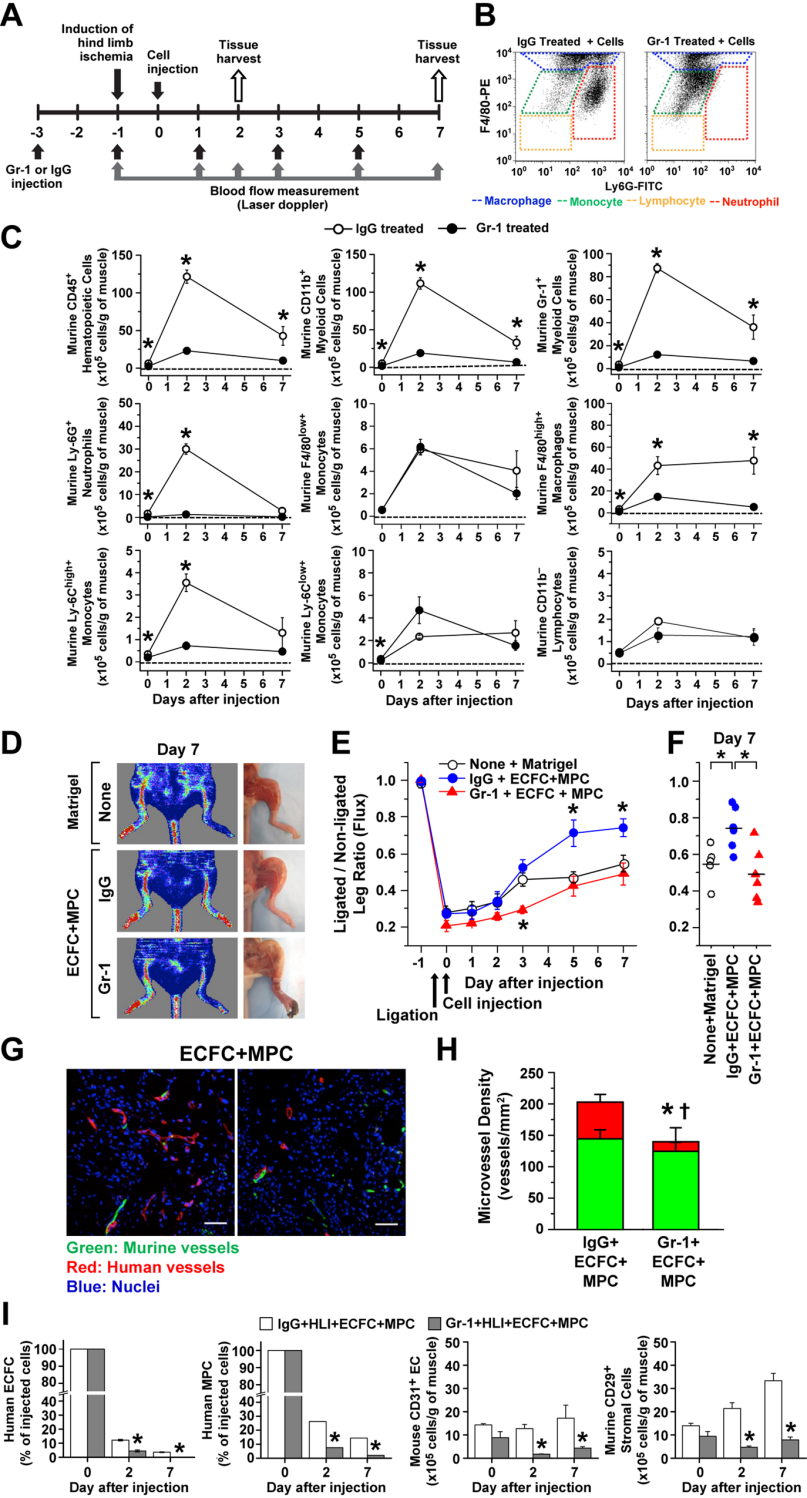
Depletion of host myeloid cells reduces blood flow recovery mediated by human ECFC + MPC in ischemic hind limb muscles. (**A**) Experimental schedule: Anti-Gr-1 or IgG control antibody (200 μg each) was given by intraperitoneal injection every 2 days. ECFC + MPC or Matrigel were injected into the ischemic hind limb muscles at day 0 (one day after hind limb ischemia induction). (**B**) Representative flow cytometry analyses of ECFC + MPC-injected ischemic hind limb muscles at day 2. (**C**) Quantitative cytometric analyses of myeloid lineage cells obtained from ischemic hind limb muscles (n = 3; means ± SEM). ^○^Shows cell number obtained from ECFC + MPC-injected ischemic hind limb muscles from mice treated with the IgG control antibody. ^●^Shows cell number obtained from ECFC + MPC-injected ischemic hind limb muscles from mice treated with anti-Gr-1 antibody. *Significant difference (P ≤ 0.05) between groups. (**D**) Representative laser Doppler images and photographs of ischemic hind limbs on day 7 after animals were injected with Matrigel or ECFC + MPC and treated with IgG control or anti-Gr-1 antibody administration. (**E**) Graph of blood flow presented as the ligated/non-ligated leg ratio (n = 5–6; means ± SEM.). *Significant difference (P ≤ 0.05) from the group of Matrigel injection with no antibody administration. (**F**) Dot graph expressed as single values for each mouse (n = 5–6; means shown by horizontal bars). *Significant difference (P ≤ 0.05) between groups. (**G**) Representative confocal images of intravenously injected lectin-labeled vessels in the ECFC + MPC-injected ischemic hind limb muscles with IgG control or anti-Gr-1 antibody administration at day 7 (Scale bars represent 50 μm). (**H**) Graph of total, human (red), and murine green) vessels in the ECFC + MPC-injected ischemic hind limb muscles with IgG control or anti-Gr-1 antibody administration at day 7 (n = 3; means ± SEM.). *Significant difference (P ≤ 0.05) between groups for total microvessel density. ^†^Significant difference (P ≤ 0.05) between groups for human microvessel density. (**I**) Quantitative cytometric analyses of human and murine vascular cells and murine stromal cells obtained from ischemic hind limb muscles at day 0, 2, and 7 (n = 3; means ± SEM). *Significant difference (P ≤ 0.05) between groups.

To elucidate whether host myeloid cells are necessary during the ECFC + MPC increased vascularization process, we depleted circulating Gr-1+ cells in mice^27^. (Gr-1 is expressed on neutrophils and monocytes.) An anti-Gr-1 or IgG control antibody was given by intraperitoneal injection every 2 days from two days before femoral artery and vein ligation to post-operative day 5 (Fig. 3A). 200 μg of anti-Gr-1 antibody per injection per mouse every 2 days was shown to deplete neutrophils and strongly reduce myeloid cells (Supplementary Fig. 4A,B). The administration of anti-Gr-1 antibody (200 μg/mouse) inhibited the recruitment of neutrophils into the ECFC + MPC injected ischemic hind limb muscle (Fig. 3B,C, Supplementary Fig. 4C). In contrast, F4/80^low+^ monocytes, Ly-6C^low+^ monocytes, and CD11b^−^ lymphocytes were not reduced at day 2 and 7 (Fig. 3C). Treatment with anti-Gr-1 antibody, compared to control IgG antibody, abolished the improved blood flow recovery seen at days 5 and 7 after ECFC + MPC injection; significantly reduced blood was measured at day 3 (Fig. 3D,E,F). Total perfused vessel density was reduced significantly with anti-Gr-1 antibody compared to IgG antibody (202.80 ± 12.20/mm^2^ vs. 173.40 ± 98.13/mm^2^, p < 0.05; Fig. 3G,H). The number of human endothelial cell-lined perfused vessels formed when ECFC + MPC were injected also decreased with anti-Gr-1 antibody compared to IgG antibody (58.28 ± 14.77/mm^2^ vs. 15.24 ± 8.71/mm^2^, p < 0.05; Fig. 3G,H). Murine vessel density was similar between the two groups (144.50 ± 14.33/mm^2^ vs. 124.50 ± 14.33/mm^2^; Fig. 3H). This suggests that depletion of Gr-1-positive cells specifically reduced the ability of ECFC to participate in vessel lumen formation in the ischemic muscle.

ECFC and MPC retention was evaluated in anti-Gr-1-treated mice. Interestingly, ECFC and MPC were reduced significantly by anti-Gr-1 compared to IgG control antibody (Fig. 3I and Supplemental Table 1). In addition, the number of murine CD31^+^ endothelial cells as well as murine CD29^+^ stromal cells was also reduced by anti-Gr-1 treatment (Fig. 3I). Systemic depletion of myeloid cells suppressed the beneficial effect of blood flow recovery by ECFC + MPC injection (Fig. 3E and F), which could be due to (i) reduced retention of injected ECFC and MPC, (ii) suppressed neo-vascularization by ECFC + MPC, and (iii) decreased of angiogenesis from host endothelial cells. The reduced number of murine CD31+ endothelial cells in ischemic hind limbs of anti-Gr-1 treated animals (Fig. 3I) lends support to this third possibility. In addition, many studies demonstrate that myeloid cells contribute neo-vessel formation by paracrine mechanisms such as matrix metalloproteinases (MMP)-2 and -9^16^ and/or VEGF-A^17–19^ as well as by physical support when recruited to the perivascular sprouting site during angiogenesis.

## Discussion

In the present study we show that ECFC + MPC can form vascular networks after injection into a pathologic ischemic environment and that this results in a corresponding improvement in blood flow in the ligated limb as early as 5 days. Furthermore, we show that depletion of host myeloid cells using *in vivo* anti-Gr-1 administration diminishes the ECFC + MPC-mediated neovascularization and improved perfusion. Our results indicate that a partnership between ECFC + MPC and the host vasculature could be developed as therapeutic approach to accelerate vascular and tissue regeneration, a concept supported by our previous study in which human ECFC + MPC injected into ischemic myocardium formed functional blood vessels and mitigated adverse left ventricular remodeling after ischemia reperfusion injury in rats^28^. Further, we suggest ECFC + MPC-mediated vascular regeneration could be enhanced by coordinated delivery or enhanced recruitment of host myeloid cells.

ECFC and MPC are well-defined progenitor cells that do not express myeloid markers^3, 26, 29–32^. ECFC are highly proliferative cells *in vitro*, they have vasculogenic capability, but initially lack venous or arterial identity; they express endothelial markers^3, 26, 29–32^. MPC, which express mesenchymal markers but not endothelial markers^3, 30–32^, associate with the abluminal side of ECFC-lined vessels *in vivo* and differentiate into pericyte/smooth muscle cells^33^. In addition, MPC release angiogenic factors such as VEGF^33^, which can stimulate ECFC as well as host endothelial cells to generate new blood vessels. ﻿ECFC﻿﻿ and﻿ MPC also promote host myeloid cell recruitment^26^, which potentiates blood flow recovery. ECFC + MPC form perfused microvascular networks much more efficiently than either ECFC or MPC alone suspended in Matrigel, or other extracellular matrices, when injected sub-cutaneously into immune-deficient mice^4, 33^. Direct cell delivery, cellular homing through intravenous injection of cells capable of forming neovessels themselves, and stimulating growth from endogenous vessels have been tested exhaustively in animal models. Recent studies report functional recoveries at 14 days and longer after onset of ischemia in the murine hind limb^34–39^.

We confirmed previous findings using endothelial cells alone in that we show blood flow improved after 28 days when ECFC alone were injected (Fig. 1B). The differences between studies in terms of the time frame to see benefit of injected cells is thought to be dependent on the cell type and passage number, number of injected cells, and/or delivery schedule and route. In the present study, ECFC were injected one day after hind limb ischemia to represent a potential clinical application, which is an important difference from other studies. Delivery routes have also been compared: embryonic stem cell-derived endothelial cells showed similar neovascularization and hind limb reperfusion when intramuscular, intrafemoral artery, and intrafemoral vein injections were analyzed^40^. Further pre-clinical studies must be extensively developed in order to clarify such issues as a foundation for translation to clinical trial.

Because of the extensive cell death and tissue damage that can ensue over a one to two week time frame, it is essential to accelerate the neovascularization process in order to re-invigorate the potential of this approach for clinical application. Here we show that co-injection of human ECFC + MPC one day after femoral artery ligation resulted in a statistically significant increase in blood flow at day 5, which was sustained for at least 28 days. We attribute this to the robust vasculogenic capability of human ECFC^3, 29^, the ability of MPC to differentiate into perivascular cells^3^ and ability of ECFC + MPC to form vessels in an ischemic environment^28^. We posit that further acceleration is possible with perhaps supplementation of the cellular injection solution with endothelial survival and proliferative factors such as VEGF and basic FGF or perhaps pre-treatment of ECFC with Trichostatin A, as shown by Palii and colleagues^39^. Our experimental model presented here relied on the inherent vasculogenic potential of naïve ECFC + MPC, without supplementation or augmentation. The promising results we obtained provide a foothold from which to advance. The choice of extracellular matrix can also be improved. Matrigel may be omitted or replaced with defined extracellular matrices^4^ such as the FDA-approved synthetic peptide hydrogel, PuraMatrix. Previously, we showed that PuraMatrix enabled ECFC + MPC-mediated vessel formation as did type I collagen or fibrin matrices^4^. In summary, cell number, injection method, addition of pro-angiogenic factors, and substitution of extracellular matrix should be investigated to optimize this potential strategy.

Our study has several limitations. The first is that we have not directly tracked the human MPC by *in vivo* labeling to verify assembly into blood vessels, as done previously^33^. However, we used flow cytometry to quantify human MPC retained in the ischemic hind limbs at 2 and 7 days after cell injections (Figs 2D and 3I, Supplemental Table 1). The second is that we have not shown that the nascent human vessels per se are required for the improved blood flow recovery in the ischemic skeletal muscle. It is clear that the combination of ECFC + MPC is required, but we have not proven that the ECFC + MPC must form vessels to elicit the improved blood flow recovery. It could be that the ECFC + MPC when combined together secrete factor(s) that stimulate endogenous murine vessels to undergo neovascularization, and it is the murine vessels that are responsible for the improved blood flow recovery. The ECFC + MPC-mediated recruitment of myeloid cells could contribute to the murine neovascularization. The third is that we did not include ECFC alone and MPC alone animal groups in the myeloid cell infiltration and depletion experiments. This is justified based on results showing that injection of ECFC alone and MPC alone did not result in rapid improvement blood flow recovery (Fig. 1A–C). Improvement was seen at Day 28 but as this time frame is not practical for alleviating ischemia, the cellular mechanisms operating in these one cell models were not a focus in this part of our study. We do not rule out the possibility that host myeloid cell recruitment may occur with ECFC or MPC alone in the ischemic hind limb model. In our previous study, we showed recruitment of CD11b + myeloid cells into the subcutaneously-injected Matrigel implants with ECFC or MPC alone at day 2, although the recruited cell number appeared to be less than ECFC + MPC^26^. This suggests that ECFC or MPC alone stimulates myeloid cell recruitment, however, this is not sufficient for the vascularization to occur because there is lack of perfused human vessels in the Matrigel implants^26^.

Therapeutic regeneration of blood vessels aims to restore blood perfusion to treat pathological ischemic tissues such as peripheral vascular disease and myocardial ischemia. Several approaches have been tried for this purpose in pre-clinical and clinical studies. The administration of VEGF-A and FGF-2 to stimulate angiogenesis from the surrounding vessels toward the ischemic area has shown beneficial effects in several animal models, yet only modest and transient improvement in randomized clinical trials^41^. These confounding results may be related to the use of a single angiogenic factor, e.g. VEGF or FGF, and perhaps differences in half lives or clearance in mice versus humans^42–44^. Another consideration is the pathological state of the endothelium in humans, for example endothelial dysfunction or atherosclerosis, which may blunt pro-angiogenic stimulation. Thus, an approach that might have a greater and more persistent effect would be to administer vasculogenic cells that can build new blood vessels *in situ* as well as produce pro-angiogenic factors. Many efforts, such as microfluidic capture of ECFC from peripheral blood^45^, are under development to meet the demand for clinical trials and begin to provide patient specific ECFC. ECFC isolated from adult peripheral blood are similarly vasculogenic when combined with MPC^33^. It has also been reported that MPC isolated from human bone marrow (that we used here), white adipose tissue, skeletal muscle, and myocardium show equal capacity to modulate ECFC to form vascular networks^46^. Our studies here strongly support the further exploration of human ECFC + MPC as a strategy to restore vascular works and blood flow and thereby promote tissue regeneration.

## Methods

### Isolation and culture of human ECFC and MPC

Human umbilical cord blood was obtained from the Brigham and Women’s Hospital in accordance with an Institutional Review Board-approved protocol and according to the Declaration of Helsinki. Informed consent was not obtained as cord blood was excess human material, normally discarded, and was obtained without identifiers. ECFC were isolated from the adherent cell fraction using CD31-coated magnetic beads (Invitrogen) as described^30^. ECFC were expanded on fibronectin (FN)-coated plates (1 μg/cm^2^; Millipore, MA) using EGM-2 (without hydrocortisone; Lonza) supplemented with 20% fetal bovine serum (FBS; Hyclone) and 1x glutamine-penicillin-streptomycin (GPS; Cellgro). ECFC between passages 5 and 8 were used for all experiments. ECFC express CD31, VE-cadherin, VEGFR-2, but not CD90, CD45 or CD14^3, 31^.

MPC were isolated from the MNC fraction of human adult bone marrow (Lonza). MNC fraction isolated using Ficoll-Paque (GE Healthcare) was seeded on 1% gelatin-coated plates using MSCGM medium (Lonza) supplemented to 10% FBS, 1x GPS. Unbound cells were removed at 48 hours, and the adherent cell fraction maintained in culture until 70% confluent. MPC were expanded in MSCGM medium supplemented to 10% FBS, 1x GPS. MPC between passages 5 and 8 were used for all experiments. MPC express CD90 and CD105 but not CD31, VE-cadherin, VEGFR-2, CD45 or CD14^3, 31^.

### Mouse hind limb ischemia model

All animal experiments and procedures were conducted according to a protocol approved by the Institutional Animal Care and Use Committee at Boston Children’s Hospital in an Association for Assessment and Accreditation of Laboratory Animal Care-approved facility. All methods for mice were performed in accordance with the guidelines and regulations in the approved protocol. Immune-deficient athymic nu/nu nude mice were purchased from Massachusetts General Hospital. This mouse lacks a thymus and is thus unable to produce T cells. 18 week-old male nude mice were anesthetized with ketamine (100 mg/kg) and xylazine (10 mg/kg) intraperitoneally. After anesthesia was attained, 7-0 silk sutures were tied in the proximal and deep femoral artery and vein, and vessels between the ties were cut to block blood flow completely. The animals were administered buprenorphine after the operation and every 12 hours for 3 days. Euthanasia was carried out by CO_2_ inhalation.

#### *In vivo* vasculogenesis assay

The nude mice were allowed to recover for one day before cell injection. 2 × 10^6^ cells were suspended in 50 μL of ice-cold Phenol Red-free Matrigel (BD Bioscience, San Jose, CA), at a ratio of 2:3 (ECFC:MPC). The mice were anesthetized with ketamine (100 mg/kg) and xylazine (10 mg/kg) intraperitoneally one day after operation, and 50 μL of cell/Matrigel suspension was injected into the muscle where the femoral artery and vein ligation took place.

### Laser Doppler imaging assay

Blood flow in hind limbs was analyzed using the LDI2-IR laser Doppler blood flow imager (Moor Instruments). The imaging was performed prior to surgery and on post-operative days 0, 1, 3, 5, 7, 10, 14 and 28. The relative changes of blood flow in hind limbs were expressed as the ratio of the operated to the contra-lateral hind limb blood flow using the company software.

### *In vivo* bioluminescence assay

ECFC were infected with Lenti-pUb-fluc-GFP at a multiplicity of infection (MOI) of 10. The pUb-fluc-GFP was made based on the backbone of pHR-s1-cla. The CMV promoter was replaced by the ubiquitin promoter, followed by a firefly luciferase/GFP fusion gene^47^. Lentivirus was prepared by transient transfection of 293T cells. Briefly, pUb-fluc-GFP was cotransfected into 293T cells with HIV-1 packaging vector and vesicular stomatitis virus G glycoprotein-pseudotyped envelop vector (pVSVG). Collected supernatant was filtered using a syringe filter (0.45 μm) and concentrated by centrifuging at 5000 g for 2 hours. The virus was titrated on 293T cells. The infectivity was determined by GFP expression and luciferase/GFP-expressing ECFCs were further sorted by FACS, expanded under routine conditions.

Luciferase-expressing ECFC were suspended in 50 μL of ice-cold Phenol Red-free Matrigel in the presence or absence of MPC (Luciferase-expressing ECFC: MPC = 2:3; total 2 × 10^6^ cells). The cell suspension was injected into the muscle at the site of femoral artery ligation. On days 1, 3, 5, 7, 10, and 14 after cell injections, the entire body of each mouse was imaged using an IVIS 200 Imaging System (Xenogen Corporation). Briefly, mice were anesthetized using an isofluorane chamber and were given the substrate, luciferin (Promega), by intraperitoneal injection according to body weight (125 mg/kg). Bioluminescence was detected for 2 min after luciferin administration, and the collected data analyzed with Live Image 3.0 (Xenogen Corporation).

### *In vivo* staining by tail vein injection of UEA-I and GS-IB_4_ lectins

Rhodamine-conjugated *Ulex europaeus agglutinin*-*I* (UEA I; Vector) and *fluorescein isothiocyanate* (*FITC*)-*conjugated Griffonia simplifolia isolectin B4* (GS-IB_4_; Vector) were mixed in a 1 mM CaCl_2_- containing saline solution. The fluorescent mixture (50 μg of each lectin/100 μL/mouse) was injected intravenously and allowed to circulate for 10 min before harvesting hind limb muscle. Mice were euthanized by inhalation of CO_2_ and hind limb muscles were harvested, fixed in 10% buffered formalin overnight, incubated in 30% sucrose for another overnight, embedded in OCT, frozen, and cryosectioned (12-μm-thick sections).

### Microvessel density analysis

12 μm-thick frozen sections were mounted with Vectashield with DAPI (Vector). Perfused human or murine vessels were identified as UEA-I-labeled or GS-IB_4_-labeled lumenal structures and counted using Leica TCS SP2 Acousto-Optical Beam Splitter confocal system equipped with a DMIRE2 inverted microscope (Diode 405 nm, Argon 488 nm, HeNe 594 nm; Leica Microsystems, Germany) at room temperature. Vessel counting was performed by blinded investigators. A 40x/1.25 oil objective or a 20x/0.7 oil objective was used. Microvessel density (MVD) was analyzed with image sets using ImageJ software (NIH) and reported as vessels/mm^2^.

### Immunofluorescence staining

Murine hind limb muscles were harvested at day 2 or day 7 following cell injection, but without intravenous injection of the fluorescent lectins. Hind limb muscles were fixed in 10% buffered formalin overnight, incubated in 30% sucrose for another overnight, embedded in OCT, frozen, and cryosectioned (12-μm-thick sections). Sections were incubated with biotinylated UEA-1 (1:100) for 1 h followed by Fluorescein-Streptavidin (1:200) for 1 h at room temperature. After that, the sections were incubated with primary antibody for 1 h at room temperature. The following primary antibodies were used: rat anti-mouse CD11b antibody (1:50), rat anti-mouse Ly6G antibody (1:50), rat anti-mouse F4/80 antibody (1:100). Sections were incubated for 1 h with goat anti-rat alexa fluor-568 antibodies (1:100), and mounted with Vectashield with DAPI (Vector). Immunofluorescence was detected using Leica TCS SP2 Acousto-Optical Beam Splitter confocal system equipped with a DMIRE2 inverted microscope (Diode 405 nm, Argon 488 nm, HeNe 594 nm; Leica Microsystems, Wetzlar, Germany) at room temperature. A 40x/1.25 oil objective or a 20x/0.7 oil objective was used.

### Flow cytometry

Matrigel and the surrounding hind limb muscle were harvested from euthanized mice and enzymatically digested with Collagenase A (1 mg/mL; Roche Life Science) and Dispase (2.5 U/mL; BD Biosciences) for 2 h at 37 °C. The retrieved cells were incubated with PerCP-conjugated anti-mouse CD45 (1:100; BD Biosciences), PE-conjugated anti-mouse CD11b (1:100; BD Biosciences), PE-conjugated anti-mouse F4/80 (1:50; AbD Serotec), FITC-conjugated mLy6G (1:50; eBiosciences) and APC-conjugated Ly6C(1:100, eBioscience). Flow cytometric analyses were performed using a Guava easyCyte 6HT/2L flow cytometer (Millipore Corporation, Billerica, MA) and FlowJo software (Tree Star Inc., Ashland, OR). To determine ECFC and MPC retention, the retrieved cells were stained with PerCP-conjugated anti-mouse CD45, FITC-conjugated anti-human CD31 (1:10 BD Biosciences), and PE-conjugated anti-human CD90 (1:100 BD Biosciences). Mouse endothelial and stromal cell recruitment was stained by PerCP-conjugated anti-mouse CD45, PE-conjugated anti-mouse CD29 (1:100 eBioscience) and APC-conjugated anti-mouse CD31 (1:100 eBioscience).

### Myeloid cell depletion experiment

For myeloid cell depletion studies, either anti-mouse Ly-6G/Ly-6C (Gr-1) (herein referred to as Gr-1; Biolegend) or control (immunoglobulin G [IgG]2b; Biolegend) antibodies were administered intraperitoneally into mice every 2 days from 2 days before hind limb ischemia operation to post-operative day 5. Anti-Gr-1 given at 200 ug/mouse was shown to be sufficient to deplete neutrophils and monocytes in the circulation, confirmed by flow cytometry using phycoerythrin (PE)-conjugated Gr-1 antibody (1:100; Biolegend) (Supplementary Fig. 4A). ECFC + MPC were injected one day after femoral artery/vein ligation as described above. Blood flow in hind limbs was measured prior to the procedure and on post-operative days 0, 1, 3, 5, and 7. Hind limb muscles were harvested at day 7 for MVD analysis. In another set of experiment, hind limb muscles were harvested at days 0, 2, and 7, and digested with collagenase/dispase to prepare single cell suspensions for flow cytometry analysis.

### Statistical analyses

Values are expressed as mean ± SEM. Values were analyzed by ANOVA followed by a Fisher least significant difference posthoc test for multiple comparisons or compared using Student *t*-test for paired comparisons (OriginLab). *P* ≤ 0.050 was considered statistically significant.

## Electronic supplementary material

Supplementary Figures 1-4 and Table 1
