# Supplementary material for: Endothelial colony forming cells and mesenchymal progenitor cells form blood vessels and increase blood flow in ischemic muscle
Source: Sci Rep. 2017 Apr 10;7:770. doi: 10.1038/s41598-017-00809-1 (PMC5429692; doi:10.1038/s41598-017-00809-1)
Supplement: Supplementary file 1 — Supplementary Figures 1-4 and Table 1 [file 41598_2017_809_MOESM1_ESM.pdf]

Supplementary Figures 1-4 and Table 1 for:

Endothelial colony forming cells and mesenchymal progenitor cells form blood vessels and increase blood flow in ischemic muscle

Authors: Kyu-Tae Kang, Ruei-Zeng Lin, David Kuppermann, Juan M. Melero-Martin, Joyce Bischoff

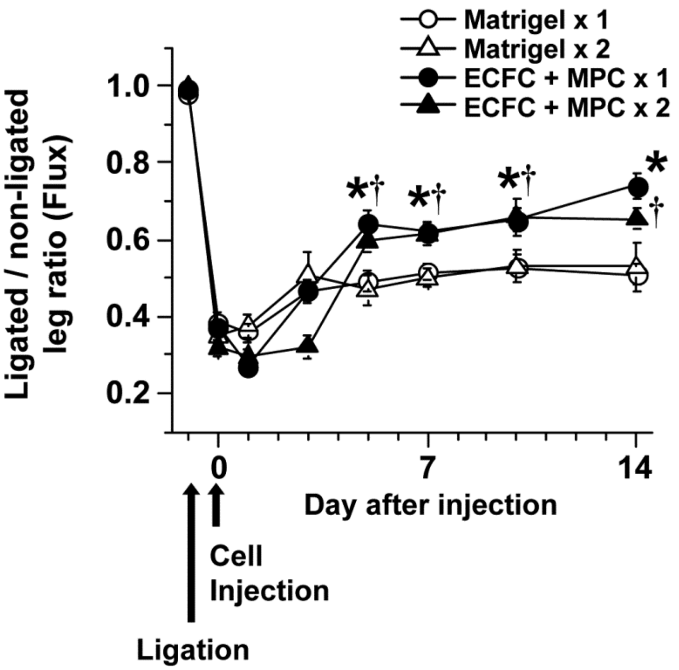

Supplementary Figure 1. Blood flow recovery over 14 days: murine ischemic hind limbs treated with x1 and x2 1human ECFC+MPC. Hind limb ischemia was induced by the ligation and cutting of femoral artery and vein at day -1. After confirmation of diminished blood flow at day 0, Matrigel, ECFC, MPC, or ECFC+MPC were injected. Injected cell number was total 2×10^6 cells suspended in 50 µL of ice-cold Phenol Red-free Matrigel. The ratio of ECFC:MPC was 2:3. In another experiment, the number of ECFC+MPC injected was increased two-fold by performing a second injection of 2×10^6 cells suspended in 50 µL of ice-cold Phenol Red-free Matrigel to investigate whether increased ECFC+MPC cell number would increase or accelerate blood flow recovery, measured by the Laser Doppler imager. Quantified graph of blood flow is presented by the igated/non-ligated leg ratio (n=6-15; means ± SEM.) to compare between x1 and x2 ECFC+MPC injection versus x1 and x2 Matrigel injection. * Significant difference ($P \leq 0.05$) between x1 ECFC+MPC and x1 Matrigel. † Significant difference ($P \leq 0.05$) between x2 ECFC+MPC and x2 Matrigel.

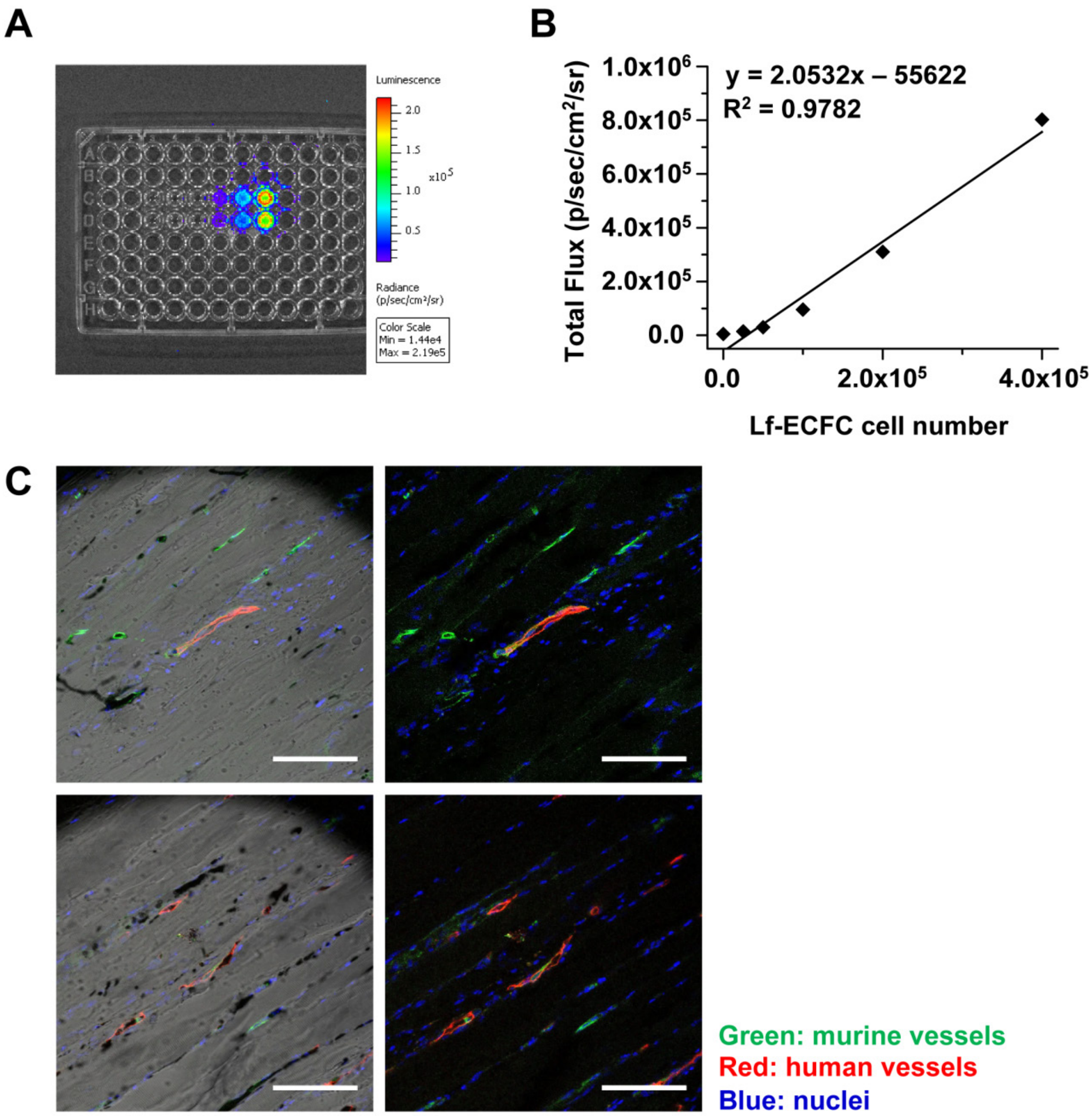

Supplementary Figure 2. Correlation between luciferase-ECFC and bioluminescence signal

Luciferase-expressing ECFC were grown, counted, and lysed. Serially diluted lysates were pipetted onto the 96 well plate. Luciferin was added to each well (final concentration = 140 μg/μL). **(A)** Representative bioluminescent signal that was detected for 2 min using an IVIS 200 Imaging System (Xenogen Corporation). **(B)** Collected data were analyzed with Live Image 3.0 (Xenogen Corporation) and plotted against input Lf-ECFC cell number. **(C)** Representative confocal images show that human microvessels formed by ECFC+MPC are present in-between ischemic hind limb muscle fibers (Scale bars represents 100 μm).

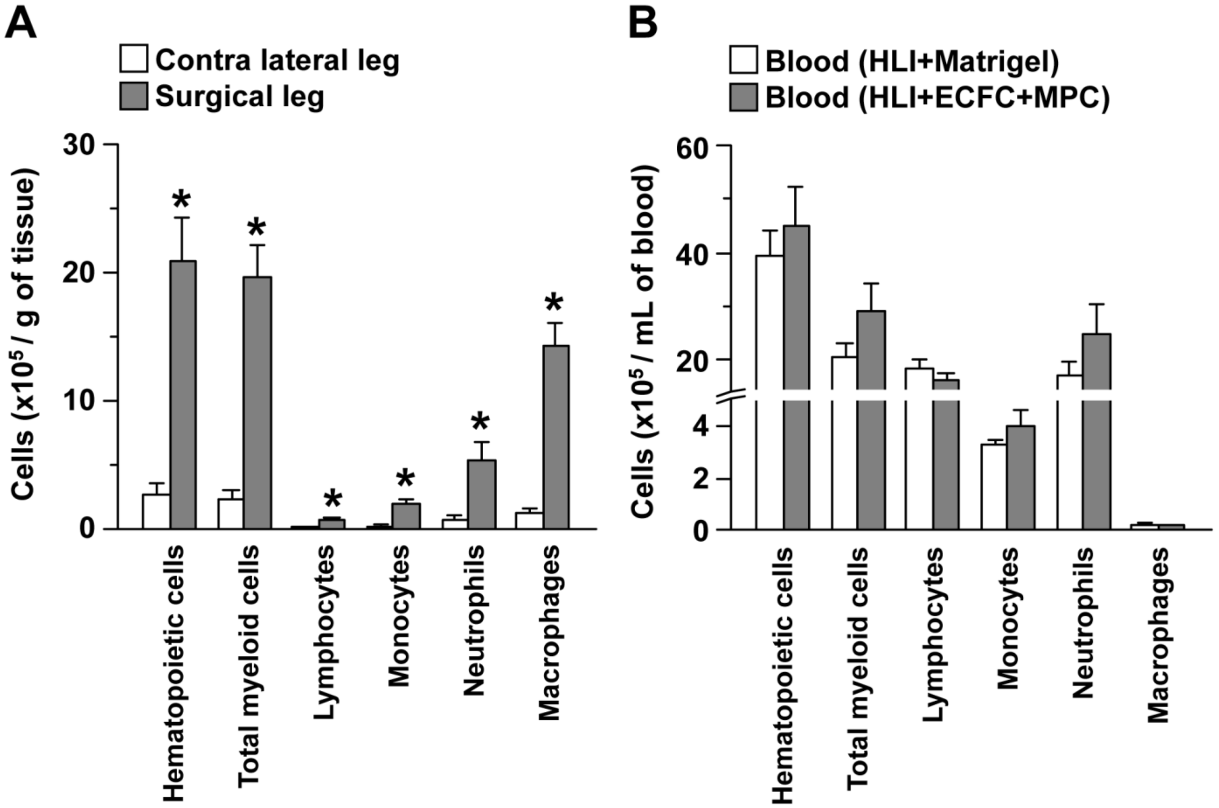

Supplementary Figure 3. Flow cytometric analysis of the myeloid cells in the ischemic hind limb muscle or peripheral blood (A) Hind limb muscle was obtained 2 days after femoral artery/vein ligation, without any cell injection. Flow cytometric analysis was performed to determine if the recruitment of myeloid lineage cells is dependent on the ischemic condition. $CD45^+$ hematopoietic cells, $CD11b^-$ lymphocytes, $CD11b^+$ myeloid cells and myeloid lineage cells including Ly-$6G^+$ neutrophils, $F4/80^{low+}$ monocytes, and $F4/80^{high+}$ macrophages were increased in the ischemic hind limb muscles compared to contralateral hind limb muscles at day 2. * Significant difference ($P \leq 0.05$) between groups. **(B)** In another experiment, peripheral blood was obtained 2 days after Matrigel or ECFC+MPC injection in the ischemic hind limb. $CD45^+$ hematopoietic cells, $CD11b^-$ lymphocytes, $CD11b^+$ myeloid cells and myeloid lineage cells including Ly-$6G^+$ neutrophils, $F4/80^{low+}$ monocytes, and $F4/80^{high+}$ macrophages were similar between Matrigel and ECFC+MPC.

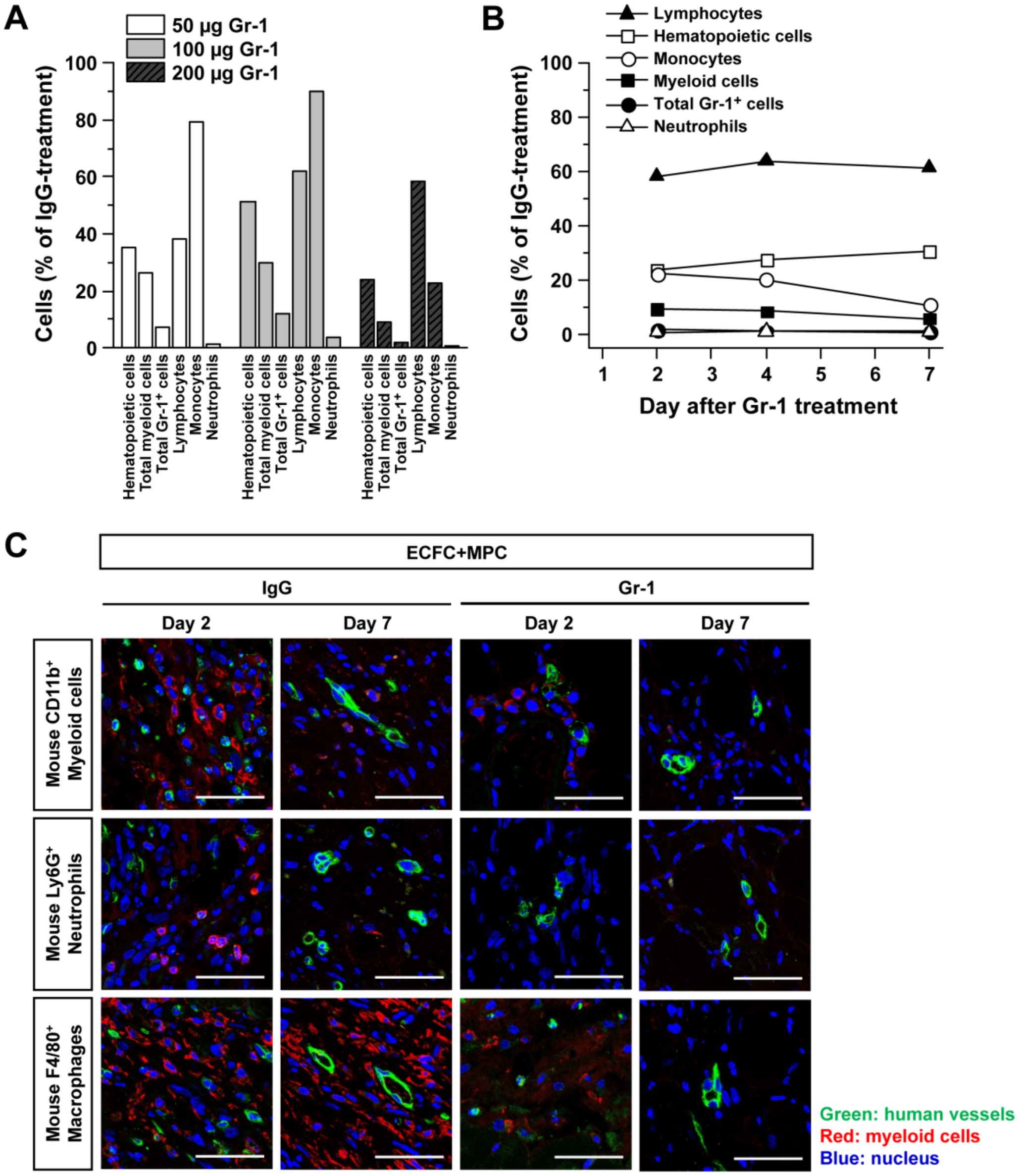

Supplementary Figure 4. Gr-1 antibody depletion of myeloid lineage cells Gr-1 antibody or control IgG antibody was administered intraperitoneally at a concentration of 50, 100, or 200 μg per mouse. Myeloid lineage cells in the blood and hind limb muscles were analyzed using flow cytometry after two days. **(A)** CD45^{+} hematopoietic cells, CD11b^{-} lymphocytes, CD11b^{+} myeloid cells, and myeloid lineage cells including Ly-6G^{+} neutrophils, F4/80^{low+} monocytes, and F4/80^{high+} macrophages, presented as a percent of control IgG antibody treated-blood sample, were reduced in a dose-dependent manner (n=2). **(B)** Administration of 200 μg of Gr-1 antibody every 2 days showed the continuous suppression of myeloid lineage cells in the ischemic hind limb muscles for 7 days. **(C)** Representative confocal images of myeloid cells in the ischemic hind limb muscles injected by ECFC+MPC with IgG or Gr-1 treatment at day 2 and 7 (Scale bars represents 50 μm).

Supplementary Table 1. Absolute cell number of engrafted ECFC and MPC within ischemic hind limb muscles (associated with Figures 2D and 3I)

**Fig.2D**	**ECFC**			**MPC**		
	Day 0	Day 2	Day 7	Day 0	Day 2	Day 7
**Cell #**	8.00 ± 0.00	0.84 ± 0.27	0.28 ± 0.08	12.00 ± 0.00	10.49 ± 3.42	1.78 ± 0.21

**Fig. 3I**	Treat-ment	**ECFC**			**MPC**		
		Day 0	Day 2	Day 7	Day 0	Day 2	Day 7
**Cell #**	IgG	8.00 ± 0.00	0.97± 0.05	0.29 ± 0.02	12.00 ± 0.00	2.10 ± 0.11	1.15 ± 0.35
**Cell #**	Anti-Gr-1	8.00 ± 0.00	0.36 ± 0.06	0.02 ± 0.00	12.00 ± 0.00	0.60 ± 0.03	0.16 ± 0.04

Each cell number presents mean ± SEM (x 10^5) from whole hind limb muscle tissue. Cell number on Day 0 is the injected cell number for ECFC and MPC.
